# Commentary on “Agreement analysis and associated factors of SARC-F and SARCCalf in screening risk sarcopenia in people living with human immunodeficiency virus”

**DOI:** 10.1016/j.clinsp.2025.100709

**Published:** 2025-07-11

**Authors:** Shubham Kumar, Ranjana Sah

**Affiliations:** a Center for Global Health Research, Saveetha Medical College and Hospital, Saveetha Institute of Medical and Technical Sciences, Saveetha University, Chennai, India; b Department of Paediatrics, Dr. D. Y. Patil Medical College Hospital and Research Centre, Dr. D. Y. Patil Vidyapeeth (Deemed-to-be-University), Maharashtra, India; c Department of Public Health Dentistry, Dr. D. Y. Patil Medical College Hospital and Research Centre, Dr. D. Y. Patil Vidyapeeth (Deemed-to-be-University), Maharashtra, India

*Dear Editor,*

We would like to commend Vieira et al.^1^ for their timely and insightful study evaluating sarcopenia risk screening tools in People Living with Human Immunodeficiency Virus (PLHIV). Their comprehensive analysis comparing the SARC-F and SARCCalf instruments addresses an important clinical challenge: early identification of sarcopenia risk in a population particularly vulnerable to muscle deterioration due to chronic infection, Antiretroviral Therapy (ART) side effects, and associated comorbidities. Given the increasing longevity of PLHIV worldwide, optimizing sarcopenia screening is a critical step toward improving long-term functional outcomes and quality of life in this population.

The authors’ methodological rigor and clear presentation of results provide valuable contributions to both clinical practice and research. Their inclusion of a wide array of sociodemographic, lifestyle, and clinical factors allows for a nuanced understanding of sarcopenia risk correlates, highlighting the multifactorial nature of muscle loss in PLHIV.^2^ Notably, their finding that the SARCCalf tool identified a higher proportion of individuals at risk than the SARC-F, with moderate agreement between the two, is particularly meaningful. This reinforces the added value of incorporating calf circumference as an indirect yet practical surrogate marker of muscle mass, which aligns with prior literature suggesting that muscle mass assessment improves sarcopenia detection sensitivity.^3^

Moreover, the authors thoughtfully contextualize their findings within the framework of socioeconomic disparities and clinical progression of HIV, illuminating how factors such as low income, smoking history, advanced disease stage, opportunistic infections, and CD4 T-cell counts contribute to sarcopenia risk.^4^ Their observations concerning the association between employment status and sarcopenia risk further underscore the importance of holistic patient assessments that encompass not only biomedical markers but also social determinants of health.

While we highly appreciate the strengths of this study, some aspects warrant further consideration to strengthen future research and clinical application. First, although the authors appropriately acknowledge the limitations inherent to their cross-sectional design, longitudinal studies would be invaluable to establishing causal relationships and understanding the temporal dynamics of sarcopenia progression in PLHIV. Such designs could clarify whether interventions targeting modifiable risk factors might alter sarcopenia trajectories.

Second, the reliance on predictive equations to estimate appendicular skeletal muscle mass, while pragmatic given resource constraints, may limit precision compared to imaging-based modalities such as DXA or MRI. Future investigations could seek to validate SARCCalf against these gold standards in PLHIV to further substantiate its diagnostic accuracy.^5^

Additionally, the relatively small sample size and the predominance of middle-aged adults restrict the generalizability of findings to older PLHIV populations, who may experience higher sarcopenia prevalence and more complex clinical scenarios. Expanding sample diversity in age and geographic representation would enhance the external validity of conclusions.

While the moderate agreement between SARC-F and SARCCalf is insightful, further research might explore whether combining these tools with objective measures such as handgrip strength or gait speed in composite screening algorithms could improve predictive performance.

We believe this study represents a significant step forward in the sarcopenia literature related to PLHIV. Its integration of clinical and social factors, combined with the comparative evaluation of two screening tools, offers actionable insights that can enhance patient care and encourage further research.

## Ethical approval

Not Applicable.

## Funding

No funding was received for this research.

## CRediT authorship contribution statement

**Shubham Kumar:** Conceptualization, Data curation, Formal analysis, Funding acquisition, Investigation, Methodology, Project administration, Resources, Software, Supervision, Validation, Visualization, Writing – original draft, Writing – review & editing. **Ranjana Sah:** Conceptualization, Data curation, Formal analysis, Funding acquisition, Investigation, Methodology, Project administration, Resources, Software, Supervision, Validation, Visualization, Writing – original draft, Writing – review & editing.

## Declaration of competing interest

The authors declare no conflicts of interest.
